# Prospective Study Evaluating a Pain Assessment Tool in a Postoperative Environment: Protocol for Algorithm Testing and Enhancement

**DOI:** 10.2196/17783

**Published:** 2020-07-01

**Authors:** Emad Kasaeyan Naeini, Mingzhe Jiang, Elise Syrjälä, Michael-David Calderon, Riitta Mieronkoski, Kai Zheng, Nikil Dutt, Pasi Liljeberg, Sanna Salanterä, Ariana M Nelson, Amir M Rahmani

**Affiliations:** 1 Department of Computer Science University of California, Irvine Irvine, CA United States; 2 Department of Future Technology University of Turku Turku Finland; 3 Department of Anesthesiology and Perioperative Care University of California, Irvine Irvine, CA United States; 4 Department of Nursing Science University of Turku Turku Finland; 5 Department of Informatics University of California, Irvine Irvine, CA United States; 6 Turku University Hospital Turku Finland; 7 School of Medicine University of California, Irvine Irvine, CA United States; 8 School of Nursing University of California, Irvine Irvine, CA United States

**Keywords:** pain measurement, pain, postoperative, acute pain, health monitoring, wearable electronic devices, machine learning, multimodal biosignals

## Abstract

**Background:**

Assessment of pain is critical to its optimal treatment. There is a high demand for accurate objective pain assessment for effectively optimizing pain management interventions. However, pain is a multivalent, dynamic, and ambiguous phenomenon that is difficult to quantify, particularly when the patient’s ability to communicate is limited. The criterion standard of pain intensity assessment is self-reporting. However, this unidimensional model is disparaged for its oversimplification and limited applicability in several vulnerable patient populations. Researchers have attempted to develop objective pain assessment tools through analysis of physiological pain indicators, such as electrocardiography, electromyography, photoplethysmography, and electrodermal activity. However, pain assessment by using only these signals can be unreliable, as various other factors alter these vital signs and the adaptation of vital signs to pain stimulation varies from person to person. Objective pain assessment using behavioral signs such as facial expressions has recently gained attention.

**Objective:**

Our objective is to further the development and research of a pain assessment tool for use with patients who are likely experiencing mild to moderate pain. We will collect observational data through wearable technologies, measuring facial electromyography, electrocardiography, photoplethysmography, and electrodermal activity.

**Methods:**

This protocol focuses on the second phase of a larger study of multimodal signal acquisition through facial muscle electrical activity, cardiac electrical activity, and electrodermal activity as indicators of pain and for building predictive models. We used state-of-the-art standard sensors to measure bioelectrical electromyographic signals and changes in heart rate, respiratory rate, and oxygen saturation. Based on the results, we further developed the pain assessment tool and reconstituted it with modern wearable sensors, devices, and algorithms. In this second phase, we will test the smart pain assessment tool in communicative patients after elective surgery in the recovery room.

**Results:**

Our human research protections application for institutional review board review was approved for this part of the study. We expect to have the pain assessment tool developed and available for further research in early 2021. Preliminary results will be ready for publication during fall 2020.

**Conclusions:**

This study will help to further the development of and research on an objective pain assessment tool for monitoring patients likely experiencing mild to moderate pain.

**International Registered Report Identifier (IRRID):**

DERR1-10.2196/17783

## Introduction

### Background

Pain is the most common reason for patients to seek medical care and is associated with many illnesses [1]. There is a high demand for tools to assess patients’ pain in the clinical context. Tools are needed especially when the patient’s own opinion is difficult to obtain. Assessment of pain is particularly difficult when the ability of a patient to communicate is limited (eg, during critical illness, in infants and preverbal toddlers, or in patients under sedation or anesthesia, with intellectual disabilities, and at the end of life) [2]. How pain is assessed and managed at the bedside is widely variable, and the prevalent practices remain suboptimal [3]. Inadequately treated pain has major physiological, psychological, economic, and social ramifications for patients, their families, and society [4]. Undertreatment of pain could result in many adverse effects and other complications and may evolve into chronic pain syndromes. It could also cause delayed discharge or prolonged recovery, which may incur higher health care costs and more patient suffering [5]. Overtreatment of pain, on the other hand, may result in unintended adverse consequences such as acute respiratory complications or in long-term complications such as opioid addiction. These issues are particularly pronounced for noncommunicative patients who are unable to articulate their experience of pain [6].

Automated and continuous pain intensity assessment for poorly communicating patients can enable timely treatment, reduce the monitoring burden on clinicians, and contribute to optimizing the use of analgesics and managing side effects and complications [7]. As pain intensity is difficult to quantify [8], the criterion standard of pain assessment is self-reporting using tools such as a visual analog scale (VAS) and numeric rating scale (NRS) [9]. These tools are rife with deficits, which are even more pronounced in vulnerable patient populations [6,10].

State-of-the-art automatic and objective pain intensity assessment techniques described in the literature use physiological data, obtained by monitoring the changes in patients’ physiological data, such as electromyography (EMG), electrocardiography (ECG), photoplethysmography (PPG), and electrodermal activity (EDA), to identify autonomic nervous system reactions to pain. One of the most well-known pain indicators is facial muscle activity. The facial nerve controls voluntary and involuntary activity of the facial muscles. The involuntary control of facial muscles is both protective and emotional, signaling the experience of pain. Other objective pain assessment tools include the surgical pleth index (formerly the surgical stress index), based on analysis of the PPG waveform and heart rate. Similarly, analysis of skin conductance has been used as a surrogate for pain in clinical situations [11].

### Objective

Wearable technology is a promising paradigm to integrate several technologies and communication solutions [12,13]. The aim of this research project is to develop an automatic and versatile pain assessment tool algorithm for detection and assessment of pain in a reliable and objective way in noncommunicative patients. The final objective of the research project is to develop a smart pain assessment tool to detect and assess pain employing behavioral and physiological indicators for a wide range of users and patients, from infants to elderly people, who are unable to communicate normally.

**Figure 1:**
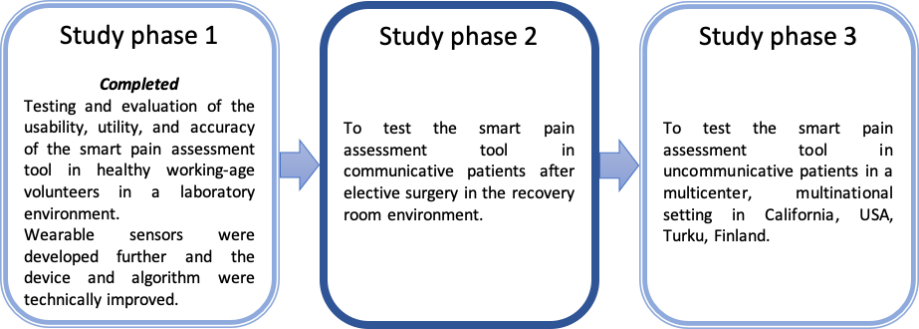
Study design for the development of a pain assessment tool.

The main research project consists of 3 study phases, which Figure 1 describes. Phase 1 of the research project focused on developing pain assessment techniques in voluntary healthy adults. In phase 1, the usability, utility, and accuracy of the new pain assessment tool were evaluated and tested in 30 healthy working-age volunteers. We used state-of-the-art standard sensors to measure bioelectrical EMG signals, as well as changes in heart rate, respiratory rate, oxygen saturation, and galvanic skin response. Based on the results, the pain assessment tool was further developed and reconstituted with modern wearable sensors, device, and algorithm.

Phase 2, described in this paper, involves the further development of and research on a pain assessment tool in patients likely experiencing mild to moderate pain. In phase 3, we will conduct a trial to assess the effectiveness of the whole platform in uncommunicative patients at 2 sites, both of which will have both intervention and control groups. In the intervention and control patients, continuous pain intensity assessments will be collected by the sensors embedded in a facial patch worn by all study patients.

## Methods

### Study Design

This protocol focuses on the second phase of a larger prospective observational data collection study to collect training data from patients likely experiencing mild to moderate pain (institutional review board [IRB] approval from Human Research Protections HS# 2017-3747). Figure 1 describes all study phases of the pain assessment tool.

The IRB approved the recruitment of 30 participants selected from the Acute Pain Service patient list at University of California, Irvine Medical Center (UCIMC) in Irvine, CA, USA. The Acute Pain Service unit at UCIMC serves over 100 participants weekly, enabling the lead physician to recruit patients for this study. We will collect primary demographic data from each patient, including height, weight, body mass index, and sex.

Approximately 30 minutes of continuous biosignals (EMG, ECG, and EDA) data will be collected from the participants. We will separate this 30-minute period into 2 parts: control (baseline pain) and experimental. Each part will consist of 2 to 3 challenge intervals in an attempt to capture pain perception before, during, and after a stimulus, with appropriate rest periods to make the statistical analysis more powerful.

In the control part, we will use a transcutaneous electrical nerve stimulation (TENS) unit [14] to obtain the patient’s baseline pain level by placing the TENS on the participant’s forearm and consistently prompting for NRS pain scores. We believe it is prudent to provide some level of baseline assessment above the patient’s existing postsurgical pain to attempt to find a baseline of pain for comparison among participants. Therefore, we are using TENS as a means of standardizing the patient threshold for experiencing pain and as a way to keep the data consistent with the previous phase of the study conducted on healthy volunteers.

In the experimental part, patients will be engaged with soft activities (eg, walking, coughing, sitting, lifting legs) that may cause a pain sensation. The participant’s experience of pain will be recorded using NRS. The NRS for pain is a unidimensional measure of pain intensity and a segmented numeric version of the VAS, in which a respondent points to a number (integers from 0 to 10) on the NRS that best represents the intensity of their pain [15]. The usual format is a horizontal bar or line. Similar to the pain VAS, the NRS is anchored by terms describing pain intensity extremes [16]. We expect to find solutions from multiple parameters that are robust in response to different acute pain cases or study designs. All protected health information will be redacted prior to data analysis.

### Participants

The prospective study will be conducted at UCIMC. The primary study population will comprise adults ranging in age from 18 to 89 years. The maximum number of patients to be asked for their consent and data collection, including withdrawals, is 30. We expect 20 to complete the study.

### Eligibility Criteria

Participants who are enrolled on this UCIMC protocol must meet the following criteria: (1) are aged 18 years or older, (2) will be consulted by the APS, (3) have the ability to communicate, (4) have provided written informed consent, and (5) have healthy, intact facial skin. Participants will be excluded if they have (1) any diagnosed condition affecting cognitive functions (eg, dementia, psychosis), (2) hand deformities that prevent the sensor from being placed, (3) any diagnosed condition affecting the central nervous system, or facial nerves or muscles, or (4) significant facial hair growth in the area where the sensors will be attached.

We must consider the natural variation in caseload (such as trauma and elective procedures) specific to both of our institutions’ regions, coupled with the fact that the recovery period is different for each patient. All candidates considered for enrollment universally will be experiencing postoperative pain during their hospitalization, and all will be receiving analgesic treatment. The study team ensures that patient safety will not be compromised while maintaining regulatory compliance, and future studies will be aimed at multicenter studies with a larger, diverse sample size to support generalizability.

### Recruitment

After IRB approval, we will screen the medical records at UCIMC, to which APS has access, to determine patient eligibility using the protocol inclusion and exclusion criteria. The anesthesiologist will approach their patients directly about study participation at the University of California Irvine Douglas Hospital, Orange, CA, USA. The study procedure will be continued if patients show interest and are suitable for the study (according to the inclusion and exclusion criteria). The study physician will explain the study in detail, providing both oral and written information. If a patient decides not to participate in the study, the study will be discontinued for this patient. If the patient is still willing to participate, the study participant enrollment log will be updated accordingly. During the study, participants’ experience of pain intensity will be recorded using the NRS.

### Informed Consent

Eligible patients will be given a consent form to consider until the following day, when the study physician will follow up regarding their participation. We encourage all participants to discuss study participation with their family and friends before consenting. Patients will be enrolled in the study only after one of the investigators has reviewed the consent form with each patient, ensuring that the patient has understood the study, has answered all questions, and has provided written informed consent. Patients are also informed about their right to withdraw from the study at any time, that their participation is voluntary, and that withdrawal would not affect their patient care.

### Clinical Trials

#### iHURT

iHURT is a system that tracks changes in the activity of facial muscles (ie, changes in facial expressions) developed at the University of Turku, Finland. It simultaneously uses physiological signs such as heart rate, heart rate variability, respiratory rate, and EDA as adjuvant measures as we attempt to develop an algorithm for pain assessment in hospitalized patients. The technology used in this study to capture the aforementioned signals includes the following components.

#### Eight-Channel Biopotential Acquisition System for EMG and ECG Recording

EMG and ECG are both biopotential signals captured from the skin surface. We developed the system used to collect them in our previous work [14]. The system includes commercially available electrodes (eg, in 24-mm diameter), electrode-to-device lead wires, an ADS1299-based portable device, and computer software (LabVIEW version 14.0f2, National Instruments) receiving streaming data from the portable device. The small analog potentials sensed from the skin surface are amplified (maximum gain: 24) and digitized, and the raw data of each channel at the rate of 500 samples per second are sent to the computer software through Bluetooth. The software visualizes the waveforms and saves the raw data into files.

**Figure 2:**
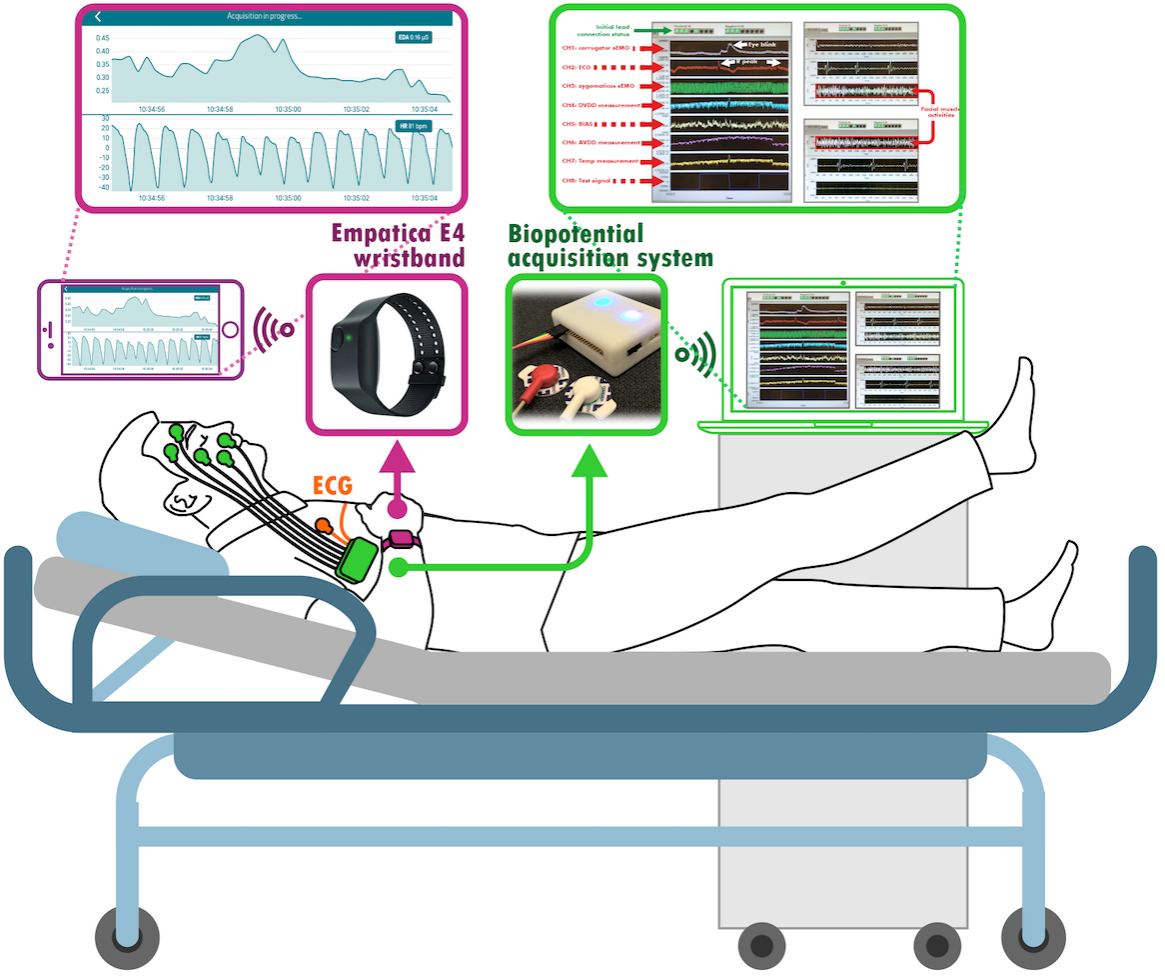
Setup of the biopotential acquisition system on the patient. ECG: electrocardiogram.

The device is configured to work in single-ended mode, called a monopolar electrode configuration, where the potential in each channel is measured between the electrode on the target site and the common reference electrode. The common reference electrode is placed on a neutral bony area behind the ear. For this reason, 2 channels are used to collect the lead I ECG, as Figure 2 illustrates. One channel measures the potential between the ECG on the right arm and the reference, and the other channel measures the potential between the ECG on the left arm and the reference. A further 5 channels are for facial EMG measurement, which monitor the activities of 5 facial muscles: frontalis, corrugator supercilii, orbicularis oculi, levator labii superioris, and zygomaticus major. The electrode placement follows the EMG research guidelines [15].

#### Empatica E4 Wristband for EDA and PPG Recording

As a sensitive and convenient measure of indexing changes in sympathetic arousal associated with emotion, cognition, and attention, EDA has been widely used in psychological studies [16] and in some commercial devices. As a noninvasive and low-cost measure of monitoring changes in blood volume, PPG has been widely used in health studies.

To monitor EDA and PPG, we use the commercially available Empatica E4 (Empatica Inc, Boston, MA, USA) wristband. The wristband will permit participants to maneuver more easily, as it will not impede their movements and will reduce the time of each patient encounter because it is considerably easier to position on the patient. The wristband has an internal memory that can record up to 36 hours of data and allows for wireless data transmission. The E4 wristband is rechargeable, with a charging time of less than 2 hours. It includes an EDA sensor, which measures the constantly fluctuating changes in certain electrical properties of the skin. This device can also noninvasively monitor blood volume pulse PPG in real time through green and red light-emitting diodes.

#### TENS Device

The TENS device is a US Food and Drug Administration (FDA)–cleared Class II over-the-counter HealthmateForever YK15AB (HealthmateForever, Lenexa, KS, USA) electrotherapy device. The TENS unit works by delivering small electrical impulses through electrodes that have adhesive pads to attach them to a person’s skin.

#### Data Collection

For patients who have consented to participate, a study team member will explain what will happen in the next 30 minutes, how to report their pain level, and what the NRS is. The team member will demonstrate how the TENS unit works on themselves with separate electrodes and explain that it is not harmful. Then, the following components will be placed on the participant: (1) the 8 leads of the EMG and ECG, (2) the Empatica E4 sensor, and (3) the TENS unit on the contralateral arm.

The biopotential acquisition device will be connected to the study analyst’s laptop and the Empatica E4 will be connected to the study analyst’s iPhone, both over Bluetooth. Data recording will begin after the study participant, clinical researcher, and study analyst are ready. The start of the recording period will be marked on the computer. Data from the sensors will be collected for a maximum period of 30 minutes. In addition, patients will be asked to complete the following parts of the procedure.

First, in the control part, after the study equipment is placed on the participant, a member of the study team will turn on the TENS device while the patient is sitting. Patients will be asked to slowly increase the TENS unit level on the contralateral arm to the highest tolerable level for them, rest for at least 10 seconds, and then decrease it to level 0, including an additional rest between TENS challenges. At each point the study team member will ask for the NRS pain score. During this period, the other devices will be simultaneously collecting physiological data.

Second, during the experimental part, as the patient is resting in the transition between the control and experimental part, the TENS unit will be disconnected. Then, patients will be engaged with soft activities (eg, walking, coughing, sitting, lifting legs) that may cause a pain sensation wearing the noninvasive devices connected excluding the TENS device. At each point (before, during, and after activity performance), the study team member will ask for the NRS pain score. During this period, the other devices will be simultaneously collecting physiological data.

Each part will consist of 2 to 3 challenge intervals in an attempt to capture pain perception using NRS before, during, and after a stimulus with appropriate rest periods to make the statistical analysis more powerful. During this period, the other devices will be simultaneously collecting physiological data.

#### Software for Data Display and Storage

We will use data acquisition software (LabVIEW version 14.0f2) to capture data from the device and sensors. As mentioned above, 2 devices will transmit data wirelessly through Bluetooth and 1 via a serial wired USB. The computer with Bluetooth USB adapters receives data from these sensor nodes. We developed a LabVIEW program in Windows for real-time waveform plotting, marking time stamps, and saving the raw data along with the time stamps. The waveform plotting function ensures checking of data validity during measurement. Raw data will be saved for offline processing and analysis.

#### Algorithm for Pain Data Analysis

The pain intensity assessment algorithm will be developed after the data collection phase, including basic signal preprocessing and a machine learning algorithm. The designed algorithm will be built based on the preliminary algorithm from phase 1 of the study and will be verified and compared between the 2 databases.

### Statistical Analysis

#### Sample Size Calculation

**Figure 3:**
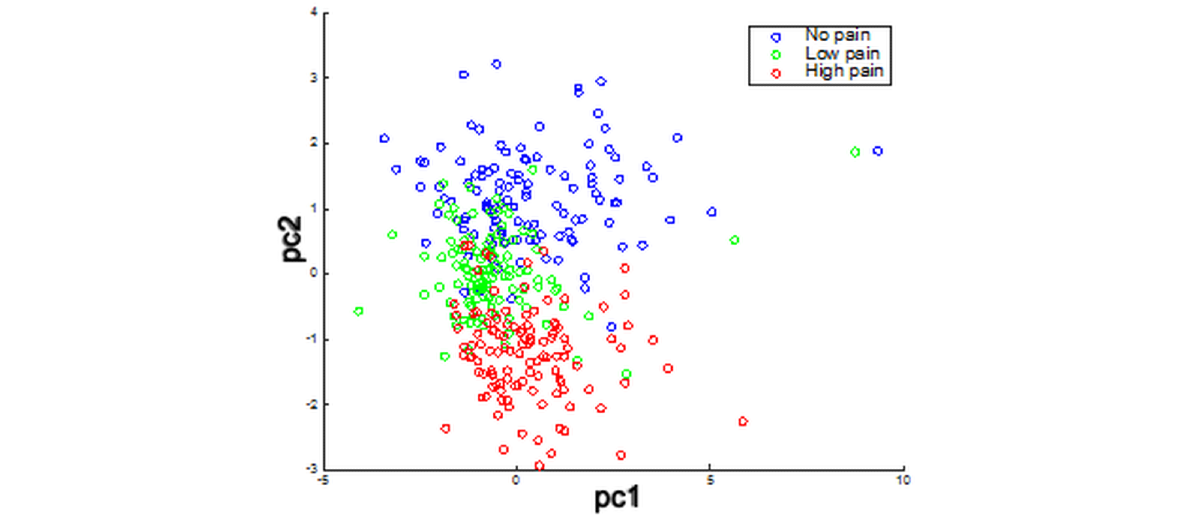
Principal component (pc) analysis of different pain levels showing all tests.

Figure 3 illustrates how multidimensional data obtained during phase 1 of the study can be processed and reduced to 2 dimensions for visualization by principal component analysis [17] to demonstrate different pain levels.

**Figure 4:**
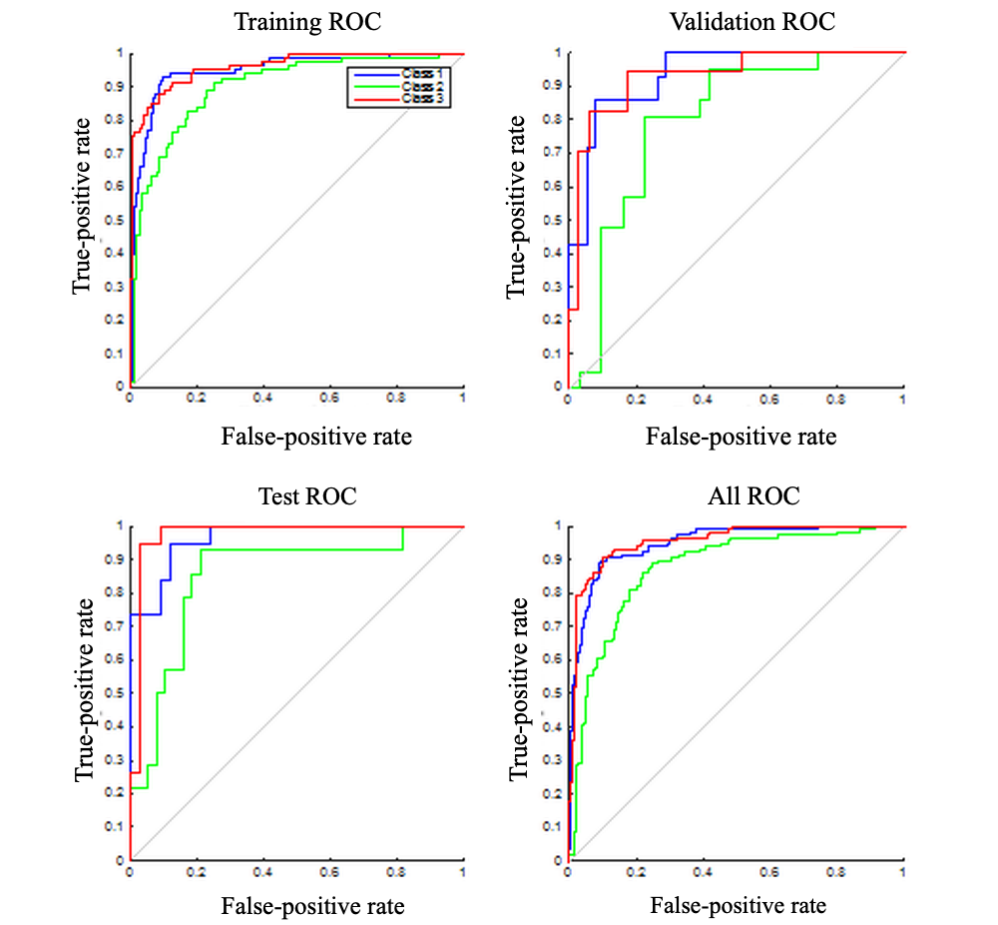
Receiver operating characteristic (ROC) curves of training, validation, and test datasets.

Figure 4 shows receiver operating characteristic curves of multidimensional data classification with an artificial neural network [18]. The results are consistent with the data visualization in Figure 3, where moderate to severe pain (shown in red) can be differentiated from no-pain samples (shown in blue). Jiang et al [19] previously published a comprehensive machine learning analysis of the phase 1 data. Our phase 1 results showed 76.7% accuracy when we used leave-1-participant-out cross-validation, showing that the data can be generalized. We also explored solutions for remote pain assessment in phase 1. We designed and implemented an electronic health system consisting of biosignal measurement and a wireless transmission device, online data processing in the cloud, and a remote data presentation webpage for the caregiver [19].

For phase 2, we will attempt to further improve the accuracy of the electronic health system and identify proper personalization methods based on the demographic data, the profile of the patient when admitted to hospital, and the biosignals collected from the patient. For the prospective study, we will recruit patients who meet the inclusion criteria. We will take into account the attrition rate and will have a maximum enrollment of 30 participants. We calculated this sample size taking into account the receiver operating characteristic analyses from the previous phase results [19,20].

#### Planned Analysis

We will design a multimodal software tool for signal processing, data fusion, and machine learning for pain assessment and classification based on the processing flow developed from study phase 1.

**Figure 5:**
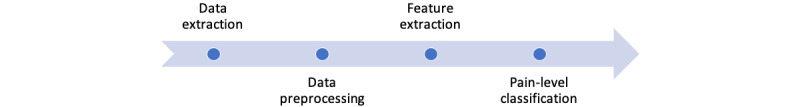
Block diagram of planned analysis.

The analysis of pain can be broken down into the following stages: data preprocessing, filtering, feature extraction, and classification (Figure 5). Different modalities collected from patients such as EMG, ECG, and EDA signals are labelled with the patient’s self-reported pain level. Once we extract these raw signals, we will use their corresponding timestamps to help synchronize the data and better understand the changes in physiological signs when pain is induced.

The next step is to digitally filter out the noise produced during data collection. Noise can be caused by multiple sources. For example, motion artifacts, baseline wander, and power channels are all common sources of noise in a clinical setting. The low- and high-frequency noise is digitally filtered using a Butterworth filter and the necessary frequencies are allowed to pass through using a bandpass filter.

For optimal classification performance, we will extract features from multiple signal domains and approaches, for example, statistical features and entropy extracted from the signal time domain and the frequency domain [20]. We will further optimize the extracted multiple features in combination to reach a balance between classification performance and computation complexity. These features are receptive to changes in pain stimuli and therefore are good factors in determining an individual’s pain levels.

Finally, once we obtain these features and their corresponding pain labels, we can use machine learning techniques to train models based on these data. Furthermore, we will automatically classify and predict the pain levels of any future patients using this existing model.

#### Outcome Variables

The final outcome of phase 2 is the prediction of patients’ pain levels based on metrics such as accuracy, sensitivity, specificity, and the area under curve with respect to patients’ self-reported NRS as the reference standard.

## Results

We have started implementing the protocol, and we are in the process of data collection. We expect to complete the analyses in early fall 2020 and then publish them. The dataset will be available for further research in early 2021.

## Discussion

### Contributions

This is, to our knowledge, the first protocol for collecting physiological signals from patients with postoperative pain for the development of an automatic pain assessment tool. Existing databases have targeted several types of pain, for example, chronic pain [21], neonatal pain [22], shoulder pain [23], or experimental acute pain [24-26], but not postoperative pain. The proposed methods and database will fill the gaps in developing and testing automatic pain assessment tools for clinical acute pain.

The proposed data collection protocol is for multimodal development, similar to some of the aforementioned databases [21,24-26]. However, it includes a variety of signal sources for development not included in existing databases, covering more than indices for autonomic nervous activities such as ECG, EDA, and PPG and nonverbal pain behaviors such as facial expressions and body postures. Thus, the proposed method could provide additional information by merging prior knowledge for each signal (eg, [27,28]) with new types of data.

### Strengths

Although the data collection focuses on postoperative pain, we included a controllable experimental pain stimulus, which was induced separate from the movement inductions. The benefit of this design is twofold. First, the physiological responses to each stimulation can be compared with each other in terms of similarity and difference within the database. Second, this design connects this study to previous studies using experimental pain stimulation only, where pain threshold (the stimulus intensity at which pain begins to be felt) and pain tolerance (the maximum pain intensity a person is able to tolerate) were mainly used as pain self-reports.

### Limitations

The main limitation is the presence of noise (or incorrect labels), making machine learning difficult. Although a certain level of noise has been shown to be positive in order to obtain a more tolerant and robust algorithm, given the real day-to-day data, the noise ratio must be low so that this does not interfere with machine learning. In our case, noise comes mainly from the cognitive difference in pain levels and motion artifacts. These artifacts can be from several sources, such as the movement of the electrodes on the face while the patient is talking.

Another limitation of this protocol is that some physiological parameter changes (eg, elevated heart rate) can result from a compound reaction to both pain stimulus and the motion itself. However, these two factors cannot be separated within the same patient, that is, moving without causing any pain. One solution could be recruiting healthy controls to make the same motions and to observe the physiological parameter response difference from postoperative patients.

### Conclusion

This study will help to further the development of and research on an objective and self-aware pain assessment tool for monitoring patients in clinical settings using both behavioral and physiological indicators. The resultant solution will be an automatic, user-centered, and versatile pain assessment system, based on the internet of things.

## Abbreviations

ECG: electrocardiography
EDA: electrodermal activity
EMG: electromyography
IRB: institutional review board
NRS: numeric rating scale
PPG: photoplethysmography
TENS: transcutaneous electrical nerve stimulation
UCIMC: University of California, Irvine Medical Center
VAS: visual analog scale
